# Cysteinyl leukotriene receptor 1 modulates retinal immune cells, vascularity and proteolytic activity in aged mice

**DOI:** 10.18632/aging.206193

**Published:** 2025-01-31

**Authors:** Andreas Koller, Julia Preishuber-Pflügl, Daniela Mayr, Susanne Maria Brunner, Anja-Maria Ladek, Christian Runge, Ludwig Aigner, Herbert Anton Reitsamer, Andrea Trost

**Affiliations:** 1 Department of Ophthalmology and Optometry, Research Program for Experimental Ophthalmology and Glaucoma Research, University Hospital of the Paracelsus Medical University, Salzburg 5020, Austria; 2 Institute of Molecular Regenerative Medicine, Paracelsus Medical University, Salzburg 5020, Austria

**Keywords:** Cysltr1, retina, proteasome activity, immune cell presence, vascular system

## Abstract

Cysteinyl leukotrienes (CysLTs) modulate the immune response, the microvasculature, cell stress and the endosomal-lysosomal system, and are involved in cellular aging. Interestingly, CysLT receptor 1 (Cysltr1) is highly expressed in the retina, a tissue that is strongly affected by the aging process. Thus, we performed an introductory examination to determine a potential importance of Cysltr1 for cells in the neurovascular unit using qPCR and immunofluorescence analysis, and on proteolytic activity in the retinas of aged mice. Aged mice (~84 weeks) were treated orally with vehicle or 10 mg/kg montelukast (MTK), a specific Cysltr1 inhibitor, for 8 weeks, 5x/week. The retinas of young mice (~11 weeks) served as controls.

Compared with young control mice, aged mice exhibited increased numbers of microglia and a reduced retinal capillary diameter, but these age-dependent changes were abrogated by MTK treatment. Retinal protein levels of the ubiquitin binding protein sequestosome-1 were amplified by aging, but were reduced by MTK treatment. Interestingly, retinal proteasome activity was decreased in aged mice, whereas Cysltr1 inhibition increased this activity.

The reduction in immune cells caused by Cysltr1 suppression may dampen neuroinflammation, a known promoter of tissue aging. Additionally, an increase in capillary diameter after Cysltr1 inhibition could have a beneficial effect on blood flow in aged individuals. Furthermore, the increase in proteolytic activity upon Cysltr1 inhibition could prevent the accumulation of toxic deposits, which is a hallmark of aged tissue. Overall, Cysltr1 is a promising target for modulating the impact of aging on retinal tissue.

## INTRODUCTION

Cysteinyl leukotrienes (CysLTs), namely, LTC_4_, LTD_4_ and LTE_4_, are lipid mediators, and as indicated by the name “leukotriene”, these ligands are primarily produced by leukocytes [1–3]. Thus, CysLTs are primarily associated with proinflammatory activity [1, 2]. Leukotrienes (LTA_4_) are derived from the arachidonic acid (AA) pathway. AA is transformed by arachidonate 5-lipoxygenase (Alox5) and arachidonate 5-lipoxygenase-activating protein (Alox5ap or FLAP) to the intermediate 5-HPETE, which is further catalyzed to LTA_4_ by LTA synthase. Later, LTC_4_ is generated by leukotriene C4 synthase [1, 4]. The three CysLT ligands act via three known G protein-coupled receptors, cysteinyl leukotriene receptor 1 (Cysltr1), Cysltr2 and 2-oxoglutarate receptor 1 (OXGR1; or CysLTE), with different affinities (Cysltr1: LTD_4_>LTC_4_; CysLTR2: LTC_4_ = LTD_4_ and CysLTE: LTE_4_) [5]. These receptors are highly expressed not only in immune cells but also in epithelial cells, endothelial cells, smooth muscle cells and diverse neuronal cells [2, 6, 7]. CysLTs are proinflammatory mediators and serve as chemoattractants and activators for immune cells such as eosinophils [2]. Furthermore, CysLT receptor activation leads to the contraction of smooth muscle cells and subsequent broncho- and vasoconstriction, and CysLTs increase vascular permeability [8]. Thus, Cysltr1 plays a pathophysiological role in asthma, and chronic treatments with Cysltr1 inhibitors such as montelukast (MTK) and zafirlukast (ZK) are used to prevent allergic reactions [8]. In addition to their immunomodulatory effects, CysLTs were shown to have proangiogenic effects, as Cysltr1 antagonists clearly promoted angiogenesis both *in vitro* and *in vivo* [9, 10]. Additionally, CysLTs and their receptors were shown to be involved in the cell stress response, especially in the oxidative stress response [7, 11–14], cell proliferation [15, 16], cell survival [14, 17] and neuronal regeneration [18].

The participation of CysLTs in aging and the pathology of age-related diseases, especially its role in chronic (neuro)-inflammation, cell stress and autophagy modulation, has been discussed in numerous publications [13, 19–23]. Recently, we showed the regulatory role of Cysltr1 in the modulation of cellular endocytosis and basal and adaptive autophagy *in vitro* using the retinal epithelial cell line ARPE-19 [7, 24, 25]. Cysltr1 inhibition led to a clear reduction in aggregated proteins *in vitro*, which highlights the potential strategy of targeting Cysltr1 activity to reduce cellular stress via autophagy induction [13]; Cysltr1 could be of interest as a target to modify cellular aging. Interestingly, we and others have shown that components of the CysLT system are highly expressed in the human, mouse and rat retina [6, 26]. In particular, the receptors Cysltr1 and Cysltr2 are highly expressed in diverse retinal layers and cell types, which highlights the need to specify the role of CysLTs in the retina.

Retinal aging is a severe problem in elderly people, as the onset and progression of age-related eye diseases can lead to vision loss, which has an enormous impact on quality of life. Thus, understanding retinal aging is of paramount importance for curing age-related diseases of the eye [27]. The connection between CysLT activity and aging or age-related disease and the presence of CysLT receptors in the retina indicate a potential role for the CysLT system in retinal aging. Thus, the main aim of this study was to identify the impact of Cysltr1 on cells of the neurovascular unit, including vascular cells, microglia, astrocytes and retinal ganglion cells (RGCs), and retinal proteolytic activity in aged mice using the specific Cysltr1 antagonist MTK.

## RESULTS

### Inhibition of Cysltr1 increases Nup62 mRNA levels

**Figure 1:**
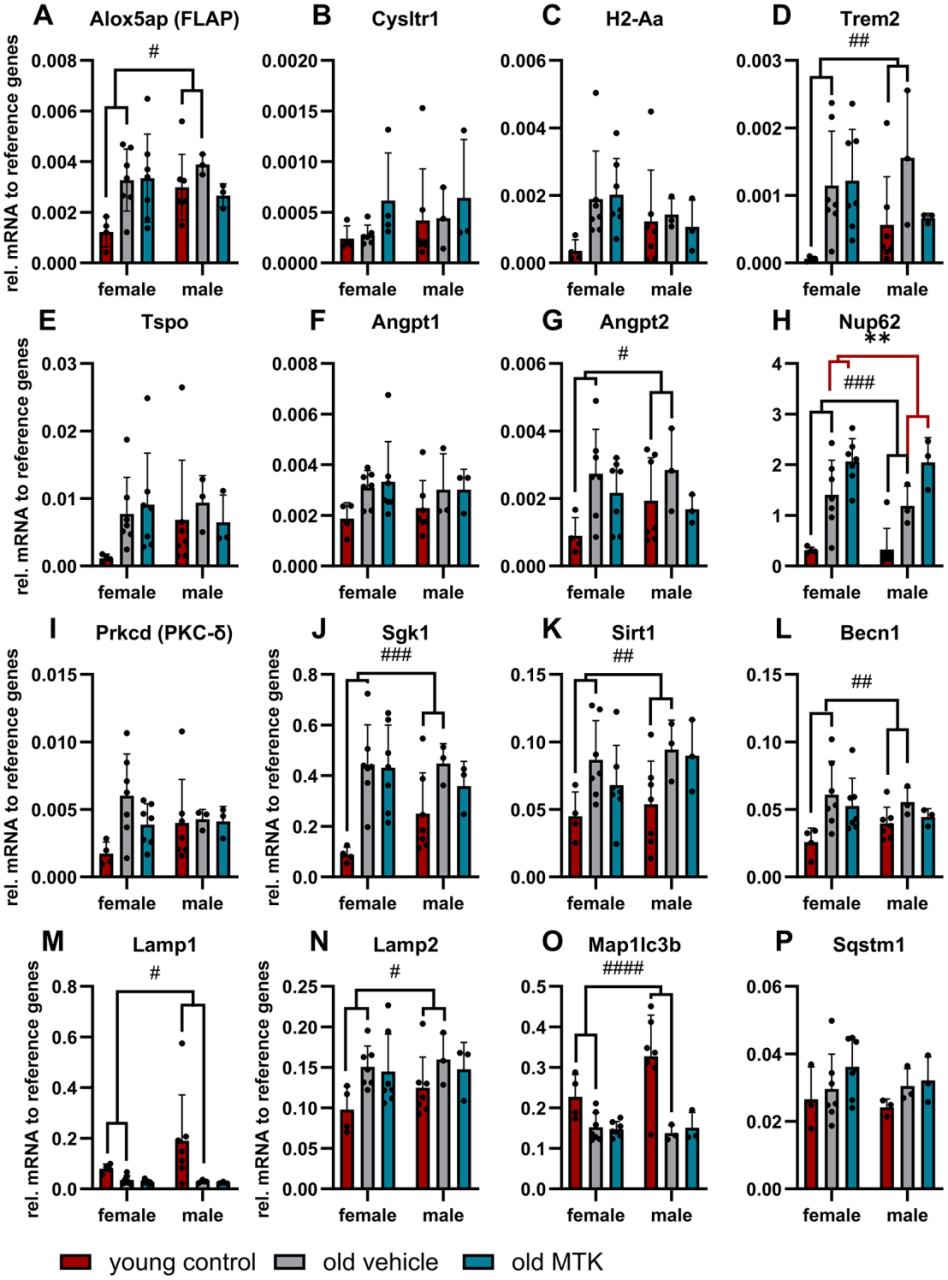
**Gene expression profile in the retinas of young untreated and vehicle- and MTK-treated old mice.** The mRNA levels of (**A**) Alox5ap, (**B**) Cysltr1, (**C**) H2-Aa, (**D**) Trem2, (**E**) TSPO, (**F**) Angpt1, (**G**) Angpt2, (**H**) Nup62, (**I**) Prkcd, (**J**) Sgk1, (**K**) Sirt1, (**L**) Becn1, (**M**) Lamp1, (**N**) Lamp2, (**O**) Map1lc3b and (**P**) Sqstm1 are represented as bar graphs and scatter plots ± SDs, *n* = 3–7. Two-way ANOVA (main factors: group and sex) followed by a Dunnett multiple comparison test. ^####^*p* < 0.0001, ^###^*p* < 0.001, ^##^*p* < 0.01, ^#^*p* < 0.05, young control group vs. old vehicle-treated group; ^**^p < 0.01, old MTK-treated group vs. old vehicle-treated group.

Initially, we verified our aging model by the expression of several genes that are reported to change with age [28] and investigated whether Cysltr1 inhibition affects the mRNA levels of these genes. The analyzed genes are associated with the leukotriene system, immune system, vascular system, cell stress response and proteolytic activity, as Cysltr1 is known to play a role in all these cellular processes. Changes in retinal gene expression due to aging and MTK treatment are summarized in Table 1. First, we quantified the mRNA levels of genes associated with the leukotriene system, namely, Alox5, Alox5ap (FLAP) and Cysltr1. Recently, we reported that in the retina, Alox5 is expressed at very low levels in specific retinal cells of the ganglion cell layer (GCL), inner nuclear layer (INL) and retinal epithelium [6]; however, the quantification of Alox5 mRNA in whole retina isolations was not possible (data not shown). Nevertheless, Alox5ap (FLAP) was quantifiable and significantly increased (main effect, *p* = 0.0340) in the retinas of aged mice compared to young mice (Figure 1A) and was unaffected by MTK treatment. Cysltr1 mRNA levels were unchanged either in the retinas of old mice or after MTK treatment (Figure 1B). Next, we analyzed genes associated with the immune system and neuroinflammation, namely, histocompatibility 2 class II antigen A alpha (H2-Aa), triggering receptor expressed on myeloid cells 2 (Trem2) and translocator protein (Tspo) [28]. Retinal Trem2 expression was significantly increased (main effect, *p* = 0.0135) in aged mice, whereas H2-Aa and Tspo were unaffected (Figure 1C–1E). MTK treatment had no effect on the three genes (H2-Aa, Trem2 and Tspo). To examine the impact of aging on the expression of genes associated with the vascular system, we analyzed angiopoietin 1 (Angpt1) and Angpt2 [28]. Retinal Angpt1 mRNA levels were not altered in the in either vehicle- or MTK-treated old animals (Figure 1F). In contrast, Angpt2 mRNA expression was increased (main effect, *p* = 0.0434) in the retinas of aged mice compared to young mice, and MTK did not affect the expression of Angpt2 (Figure 1G). As Cysltr1 is known to regulate the cellular stress response, we analyzed genes associated with cell stress and aging, namely, nucleoporin 62 (Nup62), protein kinase C delta type (Prkcd), serum/glucocorticoid regulated kinase 1 (Sgk1) and sirtuin 1 (Sirt1) genes [28]. Nup62 mRNA levels were significantly increased (main effect, *p* = 0.0004) in aged retinas and were further increased by MTK treatment (main effect, *p* = 0.0068) (Figure 1H). Neither the expression of Prkcd was affected by aging nor by treatment with MTK (Figure 1I). Sgk1 and Sirt1 were significantly upregulated (main effects, *p* = 0.0006 and *p* = 0.0116, respectively) in the aged retinas, and MTK had no effect on the expression of these genes (Figure 1J, 1K). Finally, we quantified the expression of autophagy-related genes, namely, beclin 1 (Becn1), lysosomal-associated membrane protein 1 (Lamp1), Lamp2, microtubule-associated proteins 1A/1B light chain 3B (Map1lc3b) and sequestosome-1 (Sqstm1) [28]. With the exception of Sqstm1, all of the screened autophagy-related genes were regulated by aging (Figure 1L–1P). Becn1 (main effect, *p* = 0.0144) and Lamp2 (main effect, *p* = 0.0326) mRNA levels were increased in aged retinas, whereas Map1lc3b (main effect, *p* < 0.0001) and Lamp1 (main effect, *p* = 0.0379) mRNA levels were reduced in aged retinas (Figure 1L–1P). MTK treatment had no effect on the expression of Becn1, Lamp1, Lamp2, Map1lc3b and Sqstm1 mRNA levels (Figure 1L–1P) compared to those in vehicle-treated old mice.

**Table 1:** Data summary.

***Note***		**Old mice vs. young control**		**Old mice + MTK vs. old mice**		**Female vs. Male**
		***Female***	***Male***	***Female***	***Male***	
mRNA quantification (qPCR)	Alox5ap	↑	↑	─	─	─
	Cysltr1	─	─	─	─	─
	H2-Aa	─	─	─	─	─
	Trem2	↑	↑	─	─	─
	Tspo	─	─	─	─	─
	Angpt1	─	─	─	─	─
	Angpt2	↑	↑	─	─	─
	Nup62	↑	↑	↑	↑	─
	Prkcd (PKC-δ)	─	─	─	─	─
	Sgk1	↑	↑	─	─	─
	Sirt1	↑	↑	─	─	─
	Becn1	↑	↑	─	─	─
	Lamp1	↓	↓	─	─	─
	Lamp2	↑	↑	─	─	─
	Map1lc3b	↓	↓	─	─	─
	Sqstm1	─	─	─	─	─
Protein analysis (IF)	Astrocyte area	↓	↓	─	─	Male ↓
	Microglia count - superficial	↑	↑	↓	↓	─
	Microglia count - deep	↑	↑	↓	↓	─
	Pericytes count - superficial	─	─	─	─	─
	Pericytes count - deep	─	─	─	─	─
	Capillary diameter - superficial	↓	↓	↑	↑	─
	Capillary total length - superficial	─	─	─	─	─
	Capillary branch count - superficial	↑	─	─	─	─
	Average branch length - superficial	↓	─	─	─	─
	Capillary junctions - superficial	↑	↑	─	─	─
	Capillary total length - deep	─	─	─	─	─
	Capillary branch count - deep	─	─	─	─	─
	Average branch length - deep	─	─	─	─	─
	Capillary junctions - deep	─	─	─	─	─
	RGC count - central	─	─	─	─	─
	RGC count - medial	─	─	─	─	─
	RGC count - peripheral	─	─	─	─	─
	Sqstm1 (p62) - GCL	↑	↑	↓	↓	Male ↑
	SqstM1 (p62) - INL	─	─	─	─	Male ↑
	Cathepsin D - GCL	─	─	─	─	─
	Cathepsin D - INL	─	─	─	─	─
	Lamp1 - GCL	↓	↓	─	─	─
	Lamp1 - INL	─	─	─	─	─
	Lamp2a - GCL	─	─	─	─	─
	Lamp2a - INL	↑	↑	─	─	─
	Acute DNA damage repair - GCL	─	─	─	─	─
	Acute DNA damage repair - INL	─	─	─	─	─
	Proteasome activity	↓	↓	↑	↑	─

Abbreviations: ↑: significant upregulation; ↓: significant downregulation; ─: unchanged; RGC: retinal ganglion cell; GCL: ganglion cell layer; INL: inner nuclear layer.

### MTK treatment reduces age-dependent microglia activity in the retina

**Figure 2:**
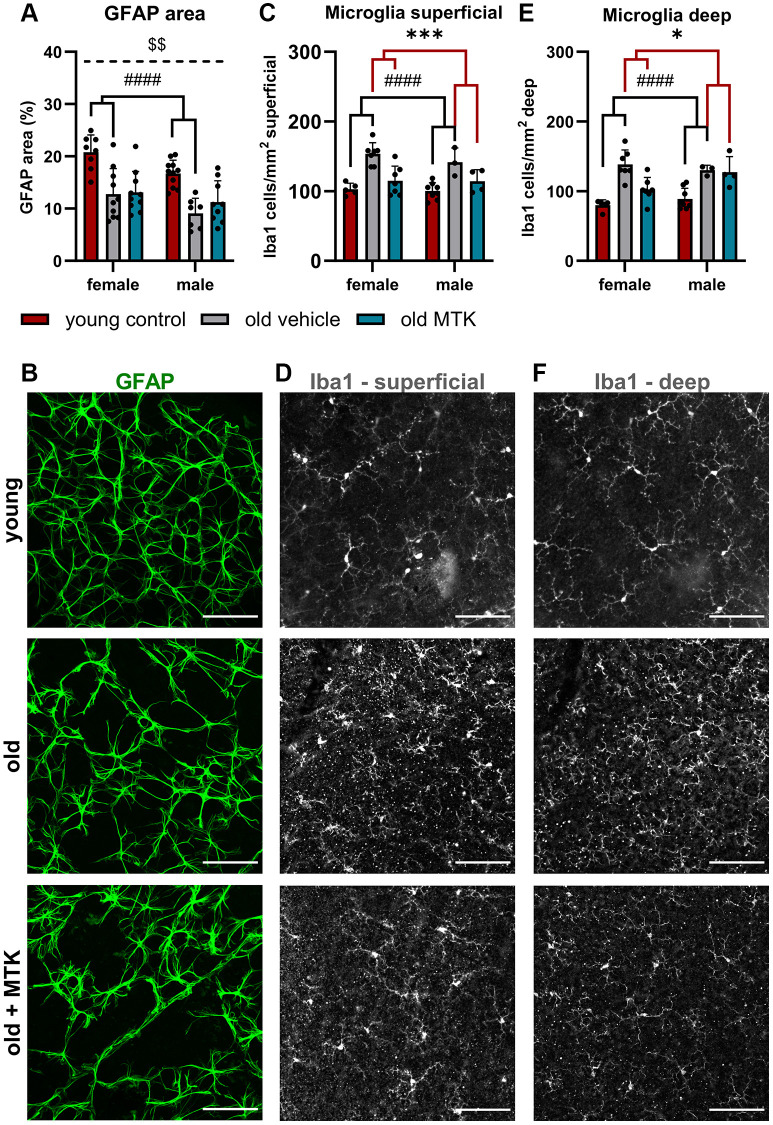
**Astrocytes and microglia were present in the retinas of young untreated and vehicle- and MTK-treated old mice.** Astrocytes were visualized by GFAP labeling and analyzed for the (**A**) positive GFAP area per image in % by ImageJ. (**B**) Representative images of GFAP-labeled (green) retinas from young untreated, vehicle-treated and MTK-treated old mice. (**C**) Microglia count and (**D**) representative Iba1 labeling (white) in the superficial retinal layers of young untreated, vehicle-treated and MTK-treated old mice. (**E**) Microglia count and (**F**) representative Iba1 labeling in the deep retinal layers of young untreated, vehicle-treated and MTK-treated old mice. Scale bar = 100 µm. The data are represented as bar graphs and scatter plots ± SDs, *n* = 3–11. Two-way ANOVA (main factors: group and sex) followed by a Dunnett multiple comparison test. ^####^*p* < 0.0001 for the young control vs. the old vehicle-treated group; ^***^p < 0.001 and ^*^p < 0.05 for the old MTK-treated group vs. the old vehicle-treated group; ^$$^*p* < 0.01 for the female vs. male group.

The proinflammatory role of Cysltr1 has been well described in the last four decades [2], but the impact of this receptor on the aging immune system is widely unknown. Therefore, we first screened retinas for the presence of retinal astrocytes, which exhibit not only homeostatic functions but also important innate and adaptive immune activities [29, 30], and of resident macrophages, namely, microglia, using immunofluorescence analysis (IF) [31]. Compared with that in young controls, the area of astrocytes in aged retinas was significantly reduced (main effect, *p* < 0.0001), and MTK treatment had no effect on the area of astrocytes in aged retinas compared to that in vehicle-treated old retinas (Figure 2A, 2B). Interestingly, we observed a sex difference (*p* = 0.0026), with male mice generally exhibiting lower retinal GFAP levels than female mice (Figure 2A). The microglial count significantly increased in the superficial (main effect, *p* < 0.0001) and deep (main effect, *p* < 0.0001) retina with age (Figure 2C–2F). Compared with vehicle treatment, inhibition of Cysltr1 reversed the aging effect and decreased the number of microglia in both the superficial (main effect, *p* = 0.0002) and deep (main effect, *p* < 0.0341) retinal layers (Figure 2C–2F).

### The age-related reduction in capillary diameter was reversed by MTK treatment

**Figure 3:**
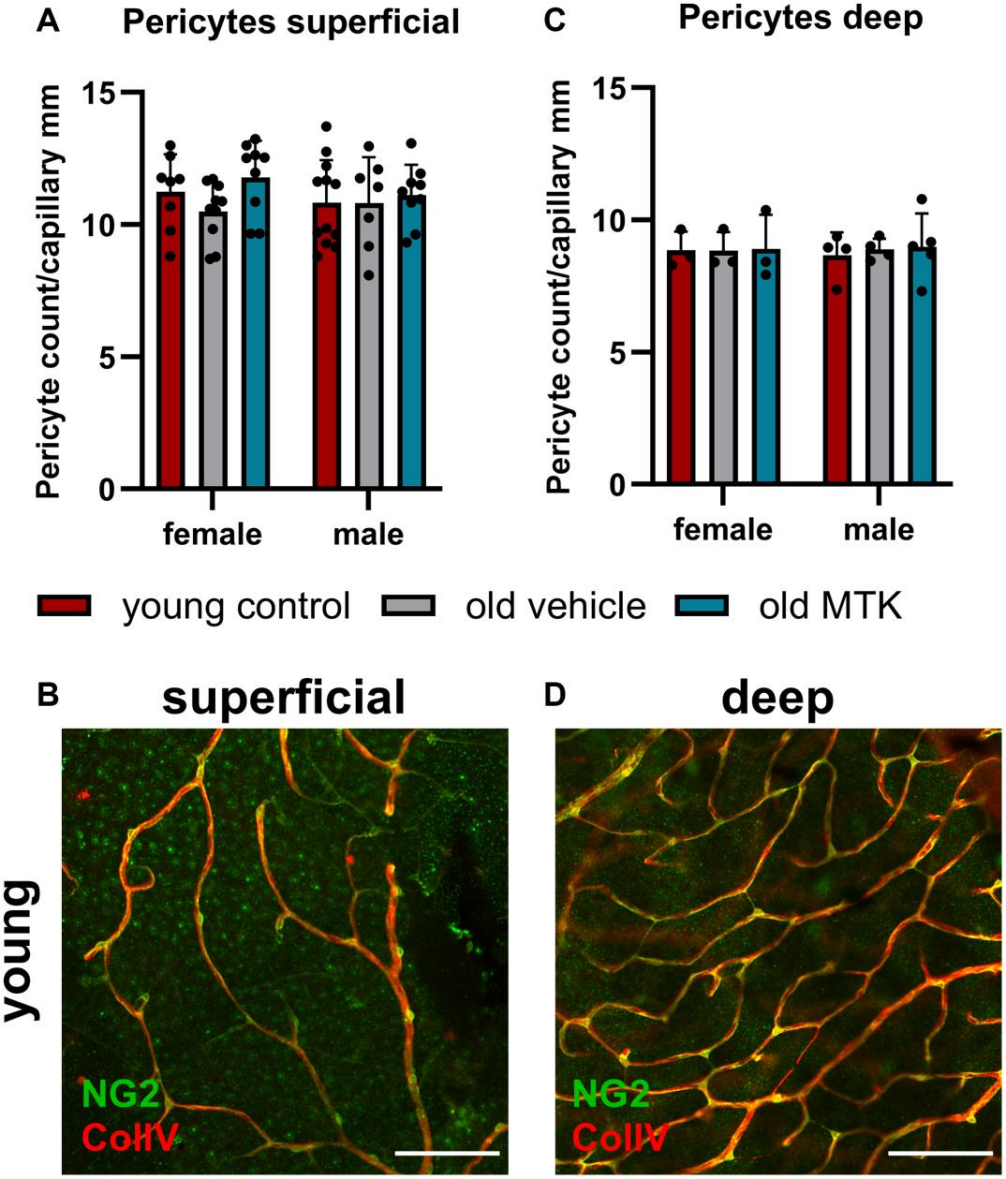
**Pericyte count per capillary mm in the retinas of young untreated, vehicle-treated and MTK-treated old mice.** The pericytes were visualized by NG2 labeling, and the capillaries were visualized by ColIV labeling. Capillary length was measured using ImageJ. (**A**) Pericyte count per capillary mm labeling in superficial retinal layers of young untreated, vehicle-treated and MTK-treated old mice and (**B**) representative NG2 (green) and ColIV (red) labeling in superficial retinal layers of young untreated mice. (**C**) Pericyte count per capillary mm in the deep retinal layers of young untreated, vehicle-treated and MTK-treated old mice and (**D**) representative NG2 and ColIV labeling in the deep retinal layers of young untreated mice. Scale bar = 100 µm. The data are represented as bar graphs and scatter plots ± SDs, *n* = 3–11. Two-way ANOVA (main factors: group and sex) followed by a Dunnett multiple comparison test.

**Figure 4:**
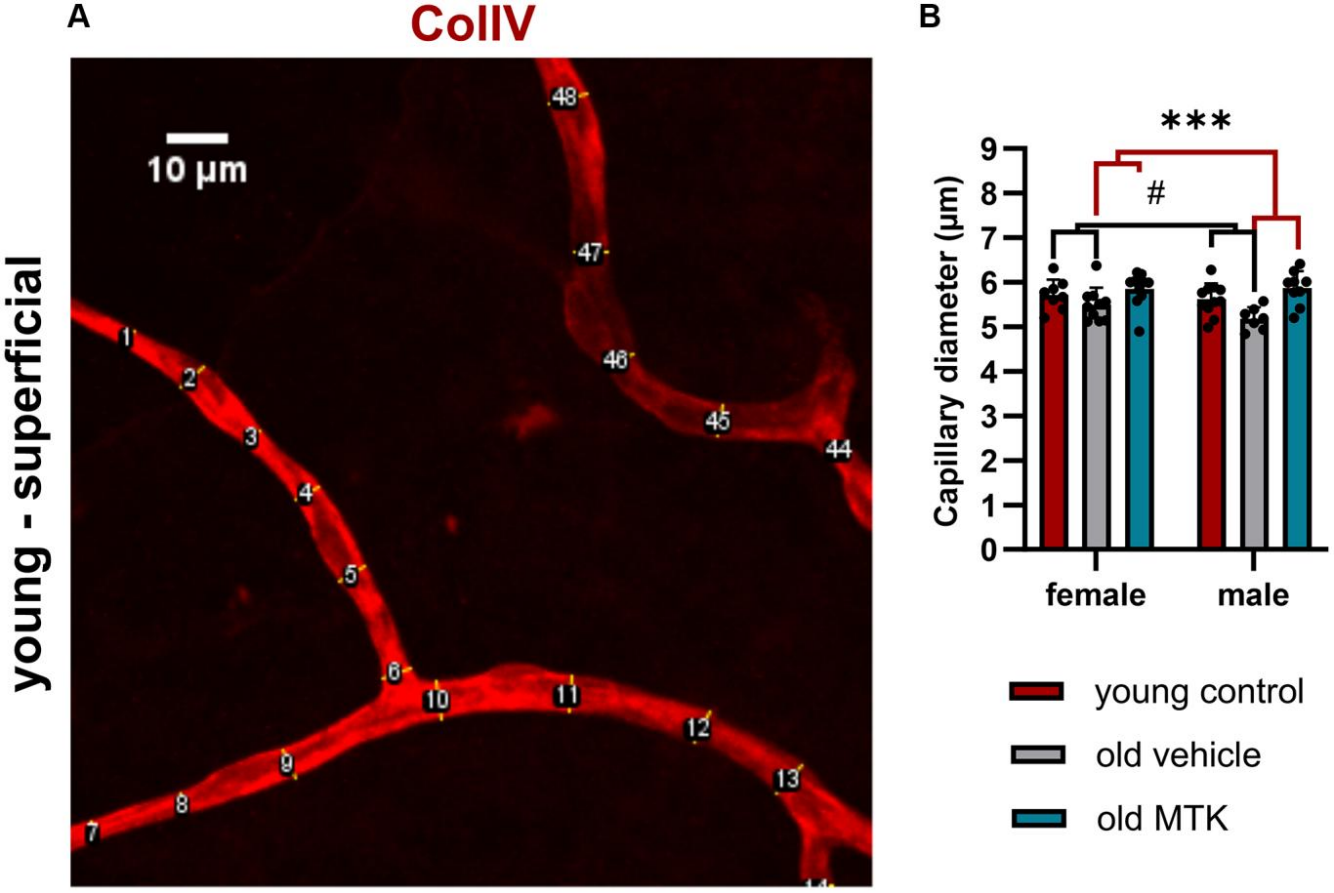
**Capillary diameter in superficial retinal layers of young untreated, vehicle-treated and MTK-treated old mice.** The capillary diameter was measured using ImageJ. (**A**) Representative image showing how the capillary diameter was measured. (**B**) Capillary diameter in the superficial retinal layers of young untreated, vehicle-treated and MTK-treated old mice. The values are represented as bar graphs and scatter plots ± SDs, *n* = 7–11. Two-way ANOVA (main factors: group and sex) followed by a Dunnett multiple comparison test. ^#^*p* < 0.05 for the young control vs. the old vehicle-treated group; ^***^p < 0.001 for the old MTK-treated group vs. the old vehicle-treated group.

**Figure 5:**
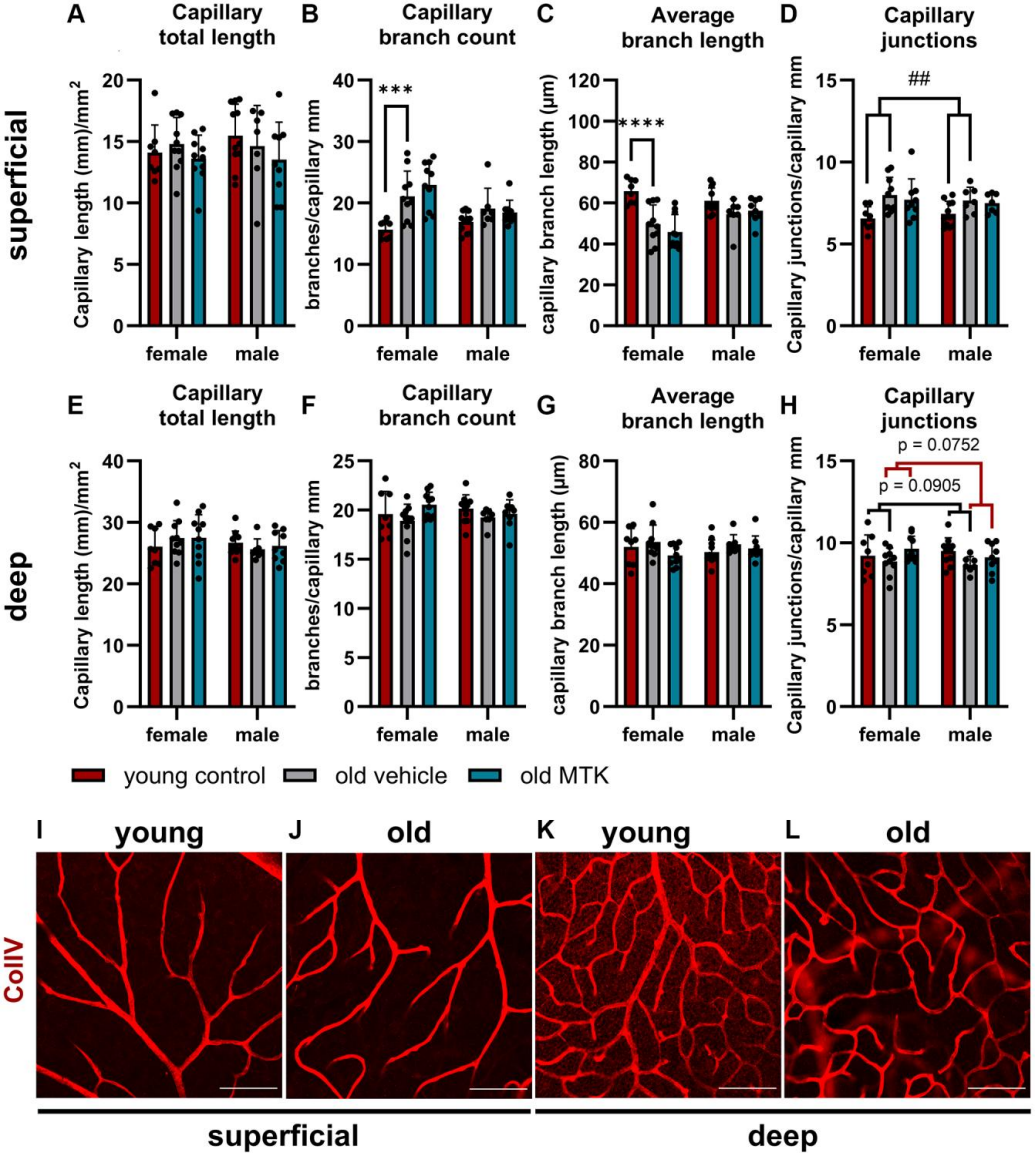
**Capillary analysis of the superficial and deep retinal layers of young untreated, vehicle-treated and MTK-treated old mice.** (**A**) Capillary total length, (**B**) branch count, (**C**) average branch length and (**D**) junctions in superficial retinal layers. (**E**) Capillary total length, (**F**) branch count, (**G**) average branch length and (**H**) junctions in deep retinal layers. Representative images of ColIV (red)-positive capillary structures in (**I**) young untreated and (**J**) old vehicle-treated superficial retinal layers and (**K**) young untreated and (**L**) old vehicle-treated deep retinal layers. Scale bar = 100 µm. The data are represented as bar graphs and scatter plots ± SDs, *n* = 7–11. Two-way ANOVA (main factors: group and sex) followed by a Dunnett multiple comparison test. ^##^*p* < 0.01 young control vs. old vehicle-treated; female-specific effect: ^****^p < 0.0001 and ^***^p < 0.001 young female control vs. old female vehicle-treated.

Cysltr1 activity has been shown to support angiogenesis and to regulate the microvasculature by increasing vessel permeability and inducing vasoconstriction [8–10]. Thus, we analyzed different parameters of the microvasculature by IF. The pericyte count per capillary mm was affected neither by aging nor by Cysltr1 inhibition (Figure 3A–3D). Although the pericyte count remained unchanged by aging, the retinal capillary diameter was significantly reduced (main effect, *p* = 0.0181) in the retinas of aged mice compared to those of young mice (Figure 4A, 4B). Interestingly, compared with vehicle treatment, Cysltr1 inhibition by MTK treatment led to a significant increase (main effect, *p* = 0.0002) in capillary diameter (Figure 4B). Next, we analyzed the overall retinal vascularity of the superficial and deep retinal layers (Figure 5A–5L). The analyzed capillaries in the superficial retinal layer showed no difference in total length among all of the experimental groups (Figure 5A) but exhibited an increased branch count (*p* = 0.007) and a decreased branch length (*p* < 0.0001) in female old mice compared to young mice, and no such difference was observed in male mice (Figure 5B, 5C). There was a sex-independent increase in the number of capillary junctions (main effect, *p* = 0.0014) in the retinas of aged mice compared to young controls (Figure 5D). MTK treatment had no effect on superficial capillaries (Figure 5A–5D). Compared with those in young controls, the number of retinal capillary junctions in the deep layer tended to decrease in old mice (*p* = 0.0905), and the number of junctions in MTK-treated animals tended to increase compared to that in vehicle-treated old mice (*p* = 0.0752) (Figure 5H). Further parameters of the deep capillary plexus, namely, capillary total length, capillary branch count and average branch length, were not significantly affected by age or by MTK treatment (Figure 5E–5G, 5K, 5L).

### CysLTR1 inhibition had no effect on retinal ganglion cell density

**Figure 6:**
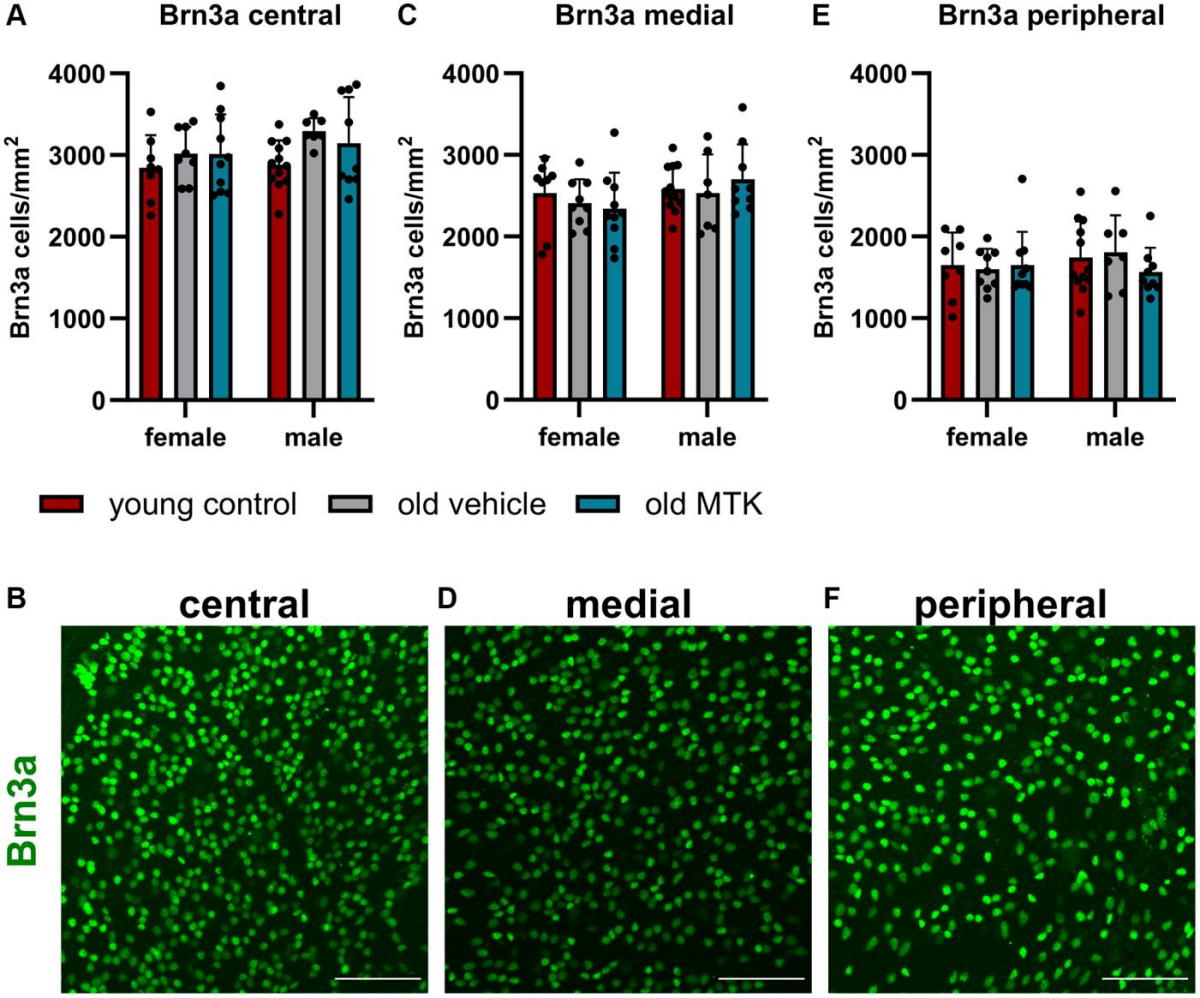
**The RGCs were located in the central, medial and peripheral regions of the retinas of young untreated, vehicle-treated and MTK-treated old mice.** RGCs were visualized by Brn3a labeling (green) and counted using ImageJ. RGC counts and representative images of Brn3a-labeled (**A, B**) central, (**C, D**) medial and (**E, F**) peripheral regions in the retinas of young untreated, vehicle-treated and MTK-treated old mice. Scale bar = 100 μm. The data are represented as bar graphs and scatter plots ± SDs, *n* = 7–11. Two-way ANOVA (main factors: group and sex) followed by a Dunnett multiple comparison test.

As Cysltr1 is known to play a role in proliferation, survival, the cell stress response and autophagy regulation, we analyzed whether Cysltr1 inhibition has an impact on the RGC count. Therefore, Brn3a was used as an RGC marker [32]. Brn3a^+^ RGCs, located in the central, medial and peripheral regions, were affected neither by aging nor by MTK treatment (Figure 6A–6F).

### MTK treatment reduced Sqstm1 protein levels, but late endosome and lysosome formation remained unchanged

**Figure 7:**
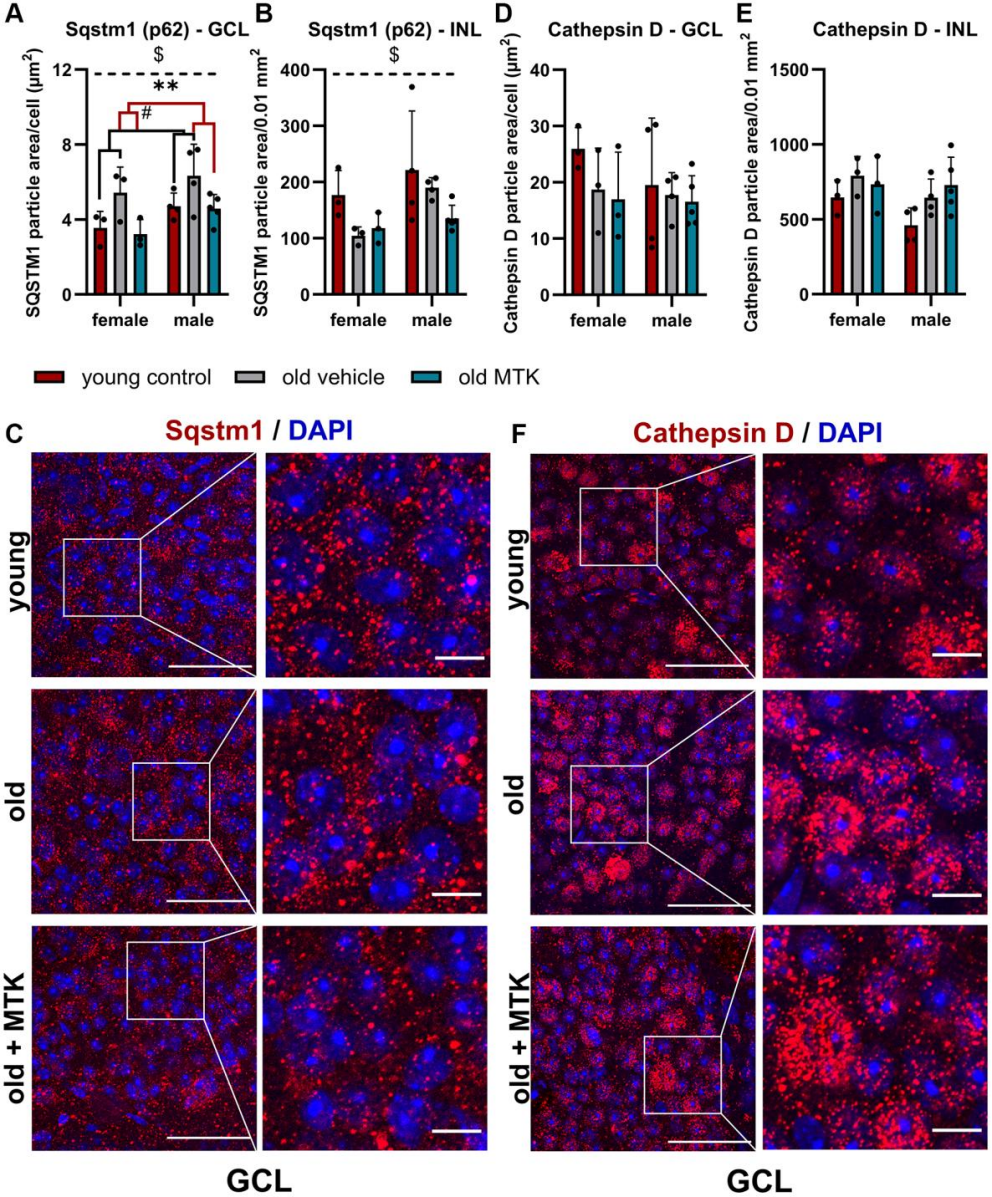
**Labeling of Sqstm1 and cathepsin D in the retinal GCL and INL of young untreated, vehicle-treated and MTK-treated old mice.** Positive labeling was analyzed using ImageJ. Sqstm1 particle area in the (**A**) GCL (area per cell) and (**B**) INL (per 0.01 mm^2^) and (**C**) representative images of Sqstm1 labeling (red) in the GCL of young untreated, vehicle-treated and MTK-treated old mice. Cathepsin D-positive area in the (**D**) GCL (area per cell) and (**E**) INL (0.01 mm^2^), and (**F**) representative images of cathepsin D labeling (red) in the GCL of young untreated, vehicle-treated and MTK-treated old mice. Scale bar images = 50 µm; scale bar images = 10 µm. The data are represented as bar graphs and scatter plots ± SDs, *n* = 3–5. Two-way ANOVA (main factors: group and sex) followed by a Dunnett multiple comparison test. ^#^*p* < 0.05 young control vs. old vehicle-treated; ^**^p < 0.01 old MTK-treated vs. old vehicle-treated; ^$^*p* < 0.05 female vs. male.

The ubiquitin binding protein Sqstm1 (p62), an important key protein for autophagic and proteasomal activity [33], has been reported to accumulate in aged tissues due to reduced autophagic activity [34]. Thus, we analyzed the protein levels of retinal Sqstm1 in the GCL and INL of aged mice using IF (Figure 7A–7C). Sqstm1 protein levels in the GCL were increased (main effect, *p* = 0.0156) in aged mice compared to young controls, but MTK treatment resulted in a significant reduction in Sqstm1 protein levels compared to those in vehicle-treated old mice (main effect, *p* = 0.0059) (Figure 7A). Aging and MTK treatment had no significant effect on Sqstm1 protein levels in the INL (Figure 7B). Interestingly, compared with female mice, male mice had significantly higher protein levels of Sqstm1 in the GCL and INL (main effects, *p* = 0.0275 and *p* = 0.0467, respectively) (Figure 7A, 7B). As a decrease in protein levels could indicate increased lysosomal activity [33], we analyzed the amount of the proteolytic enzyme cathepsin D in the GCL and INL. Cathepsin D levels remained unchanged by aging in the GCL of aged retinas and MTK treatment also had no effect on cathepsin D levels (Figure 7D, 7F). However, cathepsin D levels tended to increase (*p* = 0.1042) in the INL of aged retinas, whereas MTK had no effect on cathepsin D in the INL.

**Figure 8:**
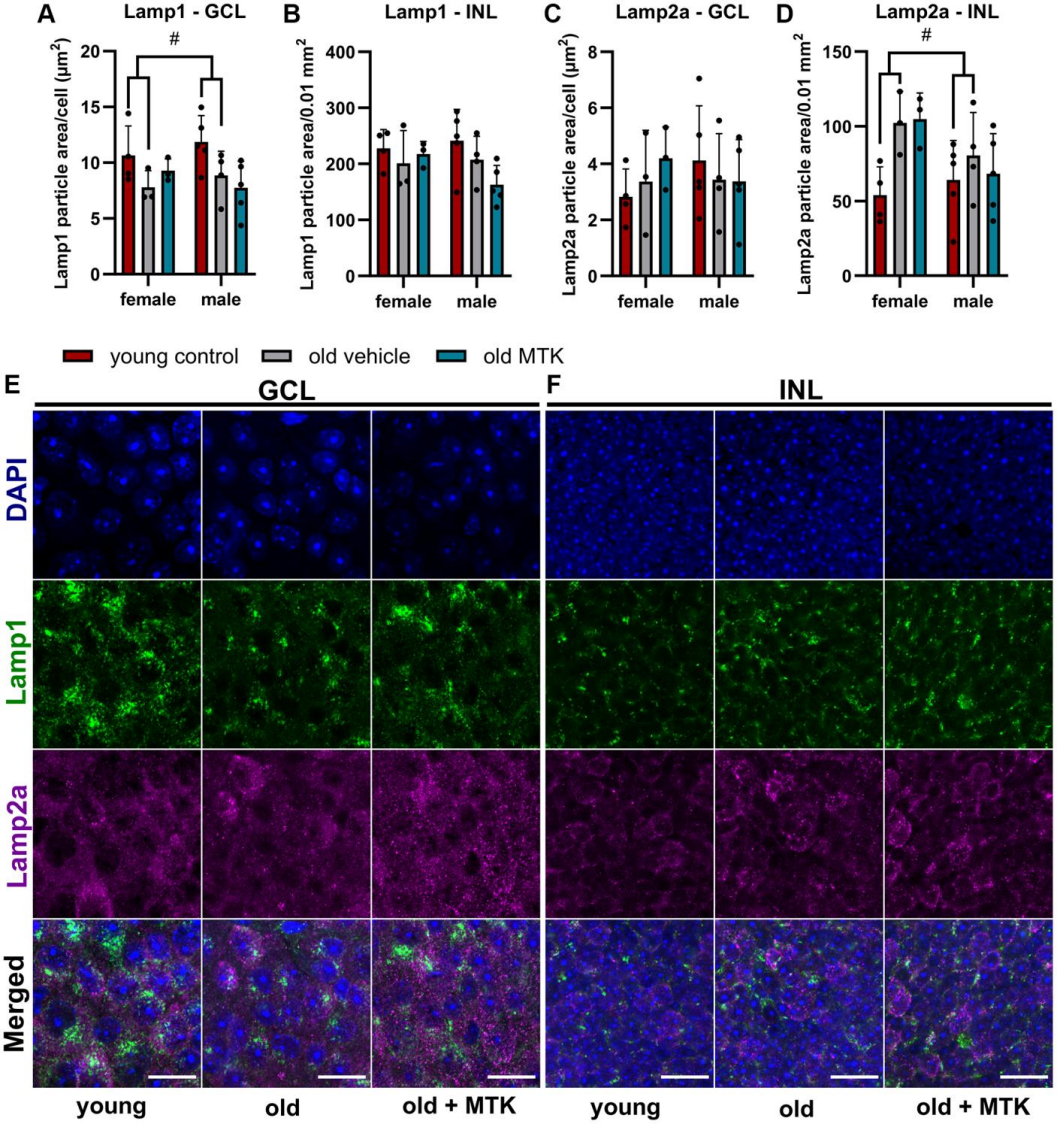
**Labeling of Lamp1 and Lamp2a in the retinal GCL and INL of young untreated, vehicle-treated and MTK-treated old mice.** Positive labeling was analyzed using ImageJ. Lamp1 particle area in the (**A**) GCL (area per cell) and (**B**) INL (per 0.01 mm^2^). Lamp2a particle area in the (**C**) GCL (area per cell) and (**D**) INL (0.01 mm^2^). Representative images of Lamp1 (green) and Lamp2a (magenta) labeling in the (**E**) GCL and (**F**) INL of young untreated, vehicle-treated and MTK-treated old mice. Scale bar images = 20 µm. The data are represented as bar graphs and scatter plots ± SDs, *n* = 3–5. Two-way ANOVA (main factors: group and sex) followed by a Dunnett multiple comparison test. ^#^*p* < 0.05 young control vs. old vehicle-treated.

Although, Lamp1 and Lamp2 mRNA levels were not affected by MTK treatment, the protein levels of Lamp1 and Lamp2a as well as late endosome and lysosome formation might be regulated by CysLTR inhibition. Therefore, we labeled and analyzed retinal Lamp1 and Lamp2a levels in the GCL and INL of aged mice using IF (Figure 8). Lamp1 protein levels in the GCL were significantly reduced (main effect, *p* = 0.0319) in old mice compared to young controls, but MTK treatment had no effect on Lamp1 protein levels (Figure 8A, 8E). However, aging and MTK treatment did not affect Lamp1 protein levels in the INL (Figure 8B). Lamp2a protein levels remained unchanged in the GCL by aging and MTK treatment (Figure 8C). Interestingly, aging significantly increased Lamp2a levels (main effect, *p* = 0.0326) in the retinal INL, whereas MTK treatment had no effect on Lamp2a levels (Figure 8D, 8F).

### Age-dependent reduction of retinal proteasome activity was enhanced after CysLTR1 inhibition

**Figure 9:**
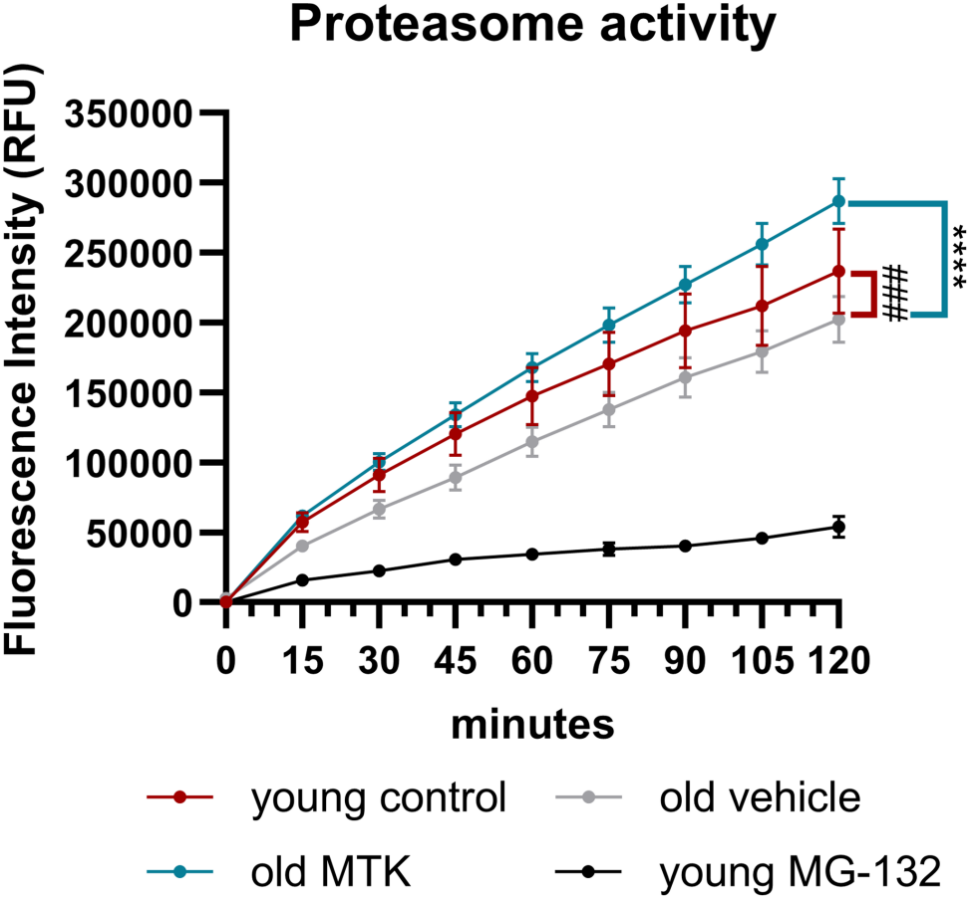
**Proteasome activity in retinal lysates of young untreated, vehicle-treated and MTK-treated old mice.** Retinal lysates were incubated for 120 minutes with the substrate Z-Gly-Gly-Leu-AMC at RT, and the fluorescence intensity (RFU) of the liberated AMC was measured every 15 minutes. MG-132 is a proteasome inhibitor and served as an assay control (*n* = 2). The values are represented as the mean ± SEM, *n* = 7–8. Two-way ANOVA (main factors: group and time) followed by a Dunnett multiple comparison test. ^###^*p* < 0.001, young control group vs. old vehicle-treated group; ^****^p < 0.0001, old MTK-treated group vs. old vehicle-treated group.

In addition, Sqstm1 is an important player in proteasome activity; therefore, we analyzed proteasome activity in the retinas of aged mice. Proteasome activity in the retinas of aged mice was significantly lower (main effect, *p* = 0.0005) than that in the retinas of young controls (Figure 9). Interestingly, compared with vehicle treatment, Cysltr1 inhibition increased proteasome activity in the retinas of aged mice (main effect, *p* < 0.0001) (Figure 9). MG-132 successfully blocked proteasome activity in retinal cell lysates (Figure 9).

### Neither aging nor CysLTR1 inhibition increased DNA damage in the mouse retina

**Figure 10:**
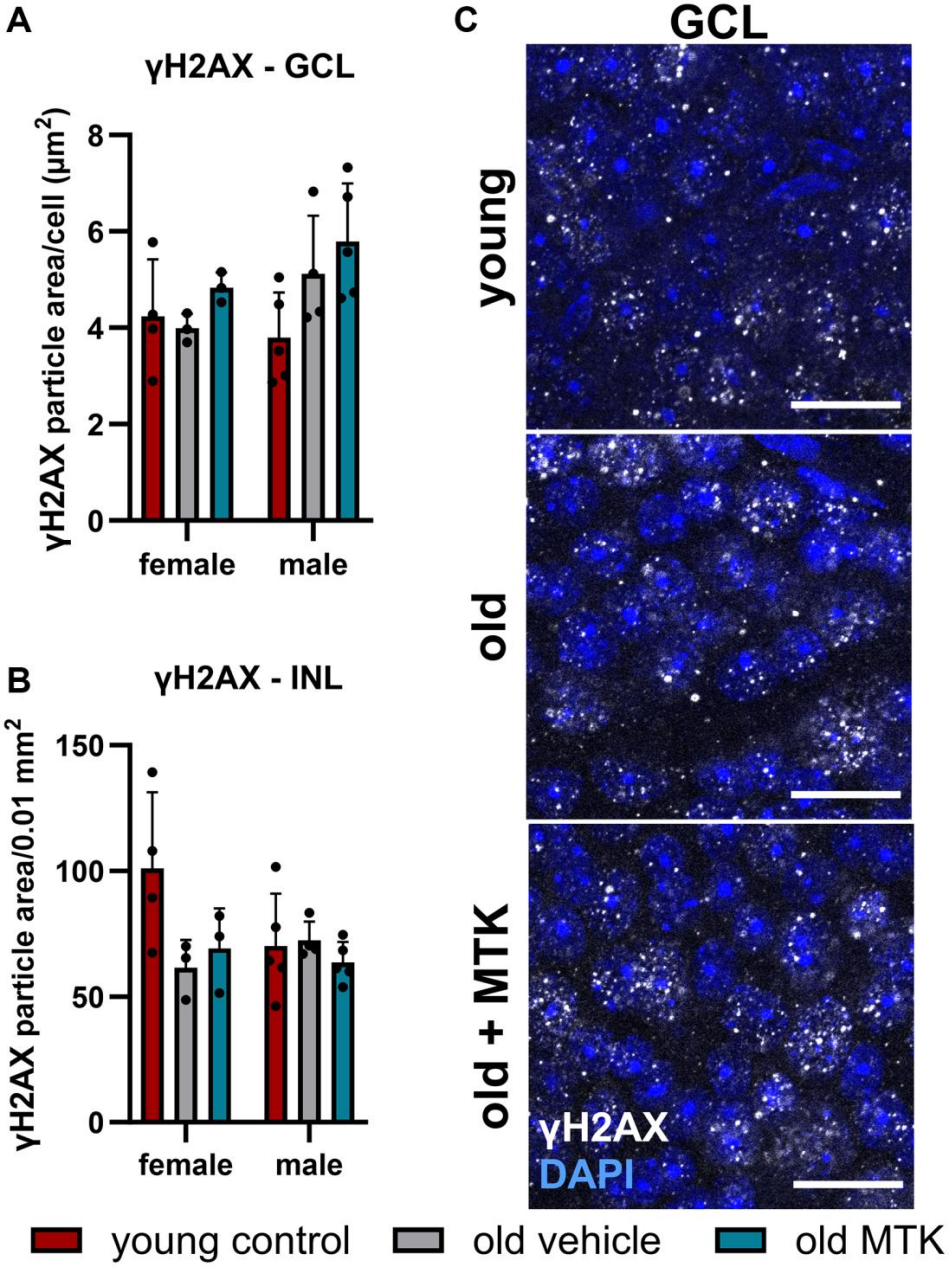
**Labeling of γH2AX in the retinal GCL and INL of young untreated, vehicle-treated and MTK-treated old mice.** Positive labeling was analyzed using ImageJ. γH2AX particle area in the (**A**) GCL (area per cell) and (**B**) INL (per 0.01 mm^2^). (**C**) Representative images of γH2AX (white) in the GCL of young untreated, vehicle-treated and MTK-treated old mice. Scale bar images = 20 µm. The data are represented as bar graphs and scatter plots ± SDs, *n* = 3–5. Two-way ANOVA (main factors: group and sex) followed by a Dunnett multiple comparison test.

The aging phenotype is associated with an increased oxidative stress [35] and increased oxidative stress might lead to elevated DNA damage. We therefore analyzed the acute double-strand breaks indirectly by quantifying the phosphorylated (Ser139) histone H2AX (γH2AX), a marker for an acute DNA damage response [36]. We observe no change of γH2AX levels in the retinal GCL and INL of aged mice and in old MTK-treated animals (Figure 10A–10C).

All data collected in this study are summarized in Table 1.

## DISCUSSION

Aging has a severe impact on retinal integrity and function, which facilitates the onset of age-related eye diseases such as age-related macular degeneration, glaucoma and diabetic retinopathy [27]. An increased understanding of the aging process in the retina will allow new strategies to prevent or cope with age-related diseases. The role of Cysltr1 in aging is part of the scientific discussion, as CysLTs are involved in diverse cellular processes, such as immunity, angiogenesis, cell stress and proteolytic activity, which participate in retinal aging [27]. An aging animal model seems to be a promising way to identify new aspects of Cysltr1-mediated functions in the context of aging. Thus, regarding Cysltr1 inhibition, long-term treatment for 8 weeks was chosen to investigate the age-dependent impact of Cysltr1 on retinal tissue. Additionally, the animals were left untreated for three days prior to retinal dissection to avoid acute responses after Cysltr1 inhibition.

Gene expression databases provide a fast overview of altered gene expression profiles in aged tissues [28]. Thus, our gene expression analysis primarily confirmed the aging phenotype in the mouse model but also provided the first evidence that Cysltr1 affects aging. The aging effect in our model was clearly observable by analyzing gene expression levels, although some genes were not significantly differentially expressed. The age-specific changes in our study are in line with reported changes in diverse tissues, such as the brain and liver [28]. However, age-dependent changes in the retinal expression of Alox5ap, Trem2, Angpt2, Nup62, Sgk1, Sirt1, Becn1, Lamp1, Lamp2 and Map1lc3b are not listed in the aging atlas database [28].

Almost daily administration of a Cysltr1 inhibitor over a period of 8 weeks did not affect the gene expression profile of the aging phenotype. Nevertheless, Cysltr1 inhibition further increased Nup62 mRNA levels. Nup62 is a part of the nuclear pore complex that is essential for the exchange of molecules between the cytoplasm and nucleus [37, 38]. Specific Nup62 activity is associated with essential physiological functions such as the transport of mRNA, stress response, cell division and maintenance of chromosomal stability [38]. Furthermore, changes in Nup62 function and levels are related to various age-related diseases including cancer, neurological diseases and rheumatoid arthritis [38]. Why Nup62 is upregulated after CysLTR1 inhibition and what possible changes occur in nuclear pore functions needs to investigated in future studies.

Sqstm1 is a ubiquitin binding protein that is essential for cellular proteostasis because it is important for the delivery of cellular waste, such as misfolded proteins, to the autophagosome and proteasome, leading to their degradation [33]. Due to the increased burden of cellular waste and the reduced efficiency of proteolytic activity in aged tissues, Sqstm1 protein accumulates in aged mice [33, 34]. Interestingly, although retinal Sqstm1 gene expression was not altered by aging or Cysltr1 inhibition, retinal Sqstm1 protein levels were increased in aged mice compared to young controls, whereas Cysltr1 inhibition reduced Sqstm1 protein levels in the retinal GCL of aged mice. The decreased protein levels of Sqstm1 indicate cellular consumption of this ubiquitin binding protein. At least one of two cellular molecule degradation processes, namely, macroautophagy and/or proteasome activity, is presumed to be more active after Cysltr1 inhibition [33].

In its processed mature form, cathepsin D is an important protease located in the lysosome [39]. Next, we analyzed cathepsin D levels in retinal tissues. Cathepsin D was not significantly affected by aging or by MTK treatment, but cathepsin D levels in the INL tented to increase by aging. These data are consistent with the increased Lamp2a levels we observed in the retinal INL of aged mice. Lamp2a is located on lysosomes and is necessary for chaperon-mediated autophagy (CMA) [33]. Thus, increased Lamp2a levels indicate an increase in CMA activity and subsequently increased lysosomal activity [33]. However, CysLTR1 inhibition had no effect on CMA as Lamp2a levels remained unchanged after MTK treatment. Using cathepsin D as a single marker to determine lysosomal activity may not be sufficient to draw specific conclusions, as cathepsin D also has nonlysosomal functions [33, 39]. Therefore, we analyzed Lamp1, a late endosomes and lysosomes marker [40] that was reduced by aging in the retinal GCL. The reduction of Lamp1 at mRNA and protein level in old animals indicates reduced biogenesis of retinal late endosomes and lysosomes, which might impact Sqstm1 levels and explain the increase of Sqstm1 protein in the GCL of old mice. However, MTK had no effect on Lamp1 levels, and late endosome and lysosome formation in retinal GCL and INL. Nevertheless, the impact of Cysltr1 inhibition on autophagy regulation *in vivo* should be analyzed in detail in future studies, especially because MTK treatment could have direct effects on autophagic activity, which were not detectable 72 hours after the last treatment, as demonstrated in the present study. Additionally, we previously observed only short-term effects of Cysltr1 inhibition on autophagy induction in RPE cells *in vitro* under basal conditions, which still resulted in a reduction in aggregated proteins [13, 24, 25].

In addition to analyzing lysosomal activity, we analyzed proteasome activity in the retinas of aged mice to further investigate the changes in Sqstm1 levels [34]. Indeed, in the retinas of aged mice, proteasome activity was reduced. In addition to the reduced Lamp1 levels, this could explain the observed increase in Sqstm1 protein levels in the GCL of aged mice, suggesting reduced proteolytic efficiency and turnover. Our data are in line with the literature, which reports a reduction in proteasome activity in diverse tissues of aged mammals [41, 42]. Most strikingly, MTK treatment increased proteasome activity, which explains the consumption and consequent reduction in Sqstm1 protein in retinal tissue cells of the GCL. The underlying mechanisms by which Cysltr1 inhibition leads to increased proteasome activity are unknown and need to be elucidated through a more detailed analysis. Nevertheless, the use of an animal model of aging further underpins the potential of Cysltr1 inhibition to manage the accumulation of cellular waste during the aging process or in age-related diseases [34, 43]. Furthermore, the higher levels of the Sqstm1 protein in male mice than in female mice should be investigated in detail in future studies.

Aging has a severe impact on immune cell activity, which is referred to as “inflammaging” [35]. Inflammaging also affects the retina, in which microglial activity is influenced by the aging phenotype [44]. Similarly, a change in the immune milieu was also observed in our experiments. The robust increase in microglial cell numbers in the retinas of aged mice suggests an increase in phagocytic and immune regulatory needs in the superficial and deep areas of the retina, especially as microglial functions, such as phagocytic capacity, decrease with age [44]. After Cysltr1 inhibition, the number of microglia in the superficial and deep capillary layers decreased significantly. On the one hand, this could be due to the anti-inflammatory properties of Cysltr1 antagonists, as CysLTs are well-known inflammatory mediators and chemoattractants [1]. On the other hand, the proteolytic activity in the retina induced by Cysltr1 inhibition could lead to a reduced accumulation of cell waste, resulting in a decreased amount of extracellular deposits and damage-associated molecular patterns (DAMPs). Accordingly, the number and activity of microglia would be decreased [44]. A detailed analysis is needed to clarify the effect of the reduced microglia count on retinal integrity in future studies.

Astrocytes have a broad spectrum of functions in the retina, including immune regulatory functions [29, 30]. In our aging model, we detected a distinct reduction in retinal coverage by astrocytes in aged mice. This observation is in line with the literature, where a reduction in retinal astrocyte count and cell spreading were observed in aged humans and rodents [45, 46]. In our initial, less detailed analysis, we did not observe an MTK-dependent effect on astrocytes; thus, a more detailed analysis is needed to identify the Cysltr1-dependent regulation of astrocyte functions.

Retinal capillaries are reportedly affected by aging; in particular, an increase in degenerated capillaries with age has been reported [47]. Furthermore, a reduction in pericyte count and coverage with age was reported in the central nervous system, but not by all studies [48]. In the peripheral rat retina, Hughes et al. did not observe age-related changes in the pericyte count per capillary length; however, they observed a decrease in the endothelial coverage of pericytes with age [49]. Data regarding pericyte count per capillary length in the context of age in the mouse retina are scarce and cannot be reasonably compared to our study, as the methods used for analysis differ. In the present study, we did not observe any changes in the retinal pericyte count per capillary mm, neither in the superficial nor in the deep capillary plexus. Furthermore, MTK did not affect the pericyte count per capillary mm. Interestingly, the capillary diameter was reduced in the retinas of aged mice compared to those of young controls, which could lead to decreased blood flow and a reduced retinal supply in aged mice [50, 51]. Cysltr1 inhibition increased the capillary diameter to a level comparable to that of young controls and could have a potential beneficial effect on the aging retina by increasing blood flow and supply. Nevertheless, we analyzed only the capillary diameter and did not measure physiological parameters. Thus, detailed experiments that include physiological measurements are needed to verify this hypothesis. Interestingly, superficial retinal vascularity was increased in old mice, as indicated by a higher branch count, shorter average branch length and greater number of capillary junctions; however, in female mice, the difference was more distinct than that in male mice. Notably, the growth factor Angp2 was upregulated in the retinas of old mice and may be related to increased superficial retinal vascularity [52]. In the deep capillary plexus, we observed reduced vascularity in the retinas of old mice compared to young controls, which was rescued by MTK treatment, although the change in capillary junctions did not reach statistical significance. However, these data are in line with the literature, as in our study, degenerated capillaries were not counted as branches, and consequently, capillary junctions were reduced [47]. In summary, our study revealed that Cysltr1 plays a critical role in vascular physiology and vascular aging in retinal tissue. Whether the modulation of the microvasculature following Cysltr1 inhibition is mediated by endothelial cells, pericytes and/or astrocytes needs to be investigated in future studies.

In this study, we did not observe a reduction in the number of Brn3a^+^ RGCs, similar to what has been discussed and described in the literature [53]. Similarly, almost daily treatment with MTK had no effect on the RGC count. Considering that daily MTK application is used for the prophylactic treatment of asthma [8], the finding that daily CysLTR1 inhibition has no effect on retinal neuron density is highly relevant. Furthermore, we did not detect increased acute DNA damage repair in retinal GCL and INL of old mice and MTK-treated old mice, suggesting that untreated and MTK-treated aged animals do not exhibit significant detrimental cellular stress in the retina.

In summary, we were able to identify new aspects of Cysltr1 functions in the retina, ranging from known properties of CysLTs such as immune regulation to completely new findings such as the regulation of proteasome activity. Although the aging phenotype was not reversed by the inhibition of Cysltr1, MTK treatment had beneficial effects on distinct aging phenotypes (microglia presence, capillary diameter and proteasome activity). However, the underlying mechanisms remain unknown. The presented data serve as a basis for new experimental set-ups to further investigate the underlying mechanisms. In particular, the regulation of proteolytic activity could be beneficial for aging cells and age-related diseases to prevent or reduce the accumulation of toxic cell waste.

## MATERIALS AND METHODS

### Tissues and tissue preparation

NG2-CreER^™^-tdTomato mice [54] were bred and housed at the animal facility of the Paracelsus Medical University and the animal facility of the Department of Ophthalmology and Optometry, Salzburg, Austria. Mice were housed for 590 days and then orally treated 5 times per week (5 days on/2 days off) at 9 am with vehicle (*n* = 17, 10 females/7 males) or 10 mg/kg MTK solution (Placebo and MTK films (IntelGenx Corporation, Quebec, Canada)) (*n* = 19, 10 females/9 males) in 25 µL H_2_O for 8 weeks. The animals were sacrificed three days after the last treatment by pentobarbital overdose. In this study, approximately 11-week-old young animals (*n* = 20, 8 females and 12 males) served as “young controls”. The exact number of animals used for each experiment can be found in the figure legend. For qPCR analysis, mouse retinas were dissected, snap frozen and stored at −80°C until further processing. For IF analysis, whole mouse eyes were fixed in 4% paraformaldehyde for 1 hour at room temperature (RT). Afterward, the eyes were washed in 100 mM phosphate buffer overnight at RT. The eyes were transferred and stored in a cryoprotective solution (0.05 M PO_4_, 25% glycerol, 25% ethylene glycol) at −20°C.

### qPCR

Mouse retinas were homogenized in 500 µl of Tri Reagent (Sigma, MO, USA). Afterward, 100 µl of chloroform was added, and the mixture was vortexed and centrifuged (12000 × g) at 4°C for 15 minutes. The aqueous phase was transferred to a new tube, and 500 µl of 96% EtOH was added. Total RNA was isolated from the mixture using a High Pure RNA Tissue Kit (Roche, Switzerland) according to the manufacturer’s protocol. Then, cDNA was synthesized using an iScript cDNA Synthesis Kit (Bio-Rad, CA, USA) according to the manufacturer’s instructions. BRYT Green dye-based GoTaq qPCR Master Mix (Promega, WI, USA), on a CFX96 system (Bio-Rad), and specific primers (listed in Table 2) were used to perform qPCR. The expression data were normalized to the levels of the reference genes Hprt, Rpl27 and Sdha (2^^-(Cq of gene of interest – mean of reference genes)^, CFX Manager Software, Bio-Rad).

**Table 2:** Primer sequences for qPCR.

	**Forward 5′–3′**	**Reverse 5′–3′**
**Alox5ap**	CAGAACTGCGTAGATGCGTA	CTCCCAGATAGCCGACAAAG
**Angpt1**	GTGGCTGCAAAAACTTGAGA	ATCTGTCAGCTTTCGGGTCT
**Angpt2**	ATGTCATCACCCAACTCCAAGA	GGATGACTGTCCACCCTCCT
**Becn1**	AAACCAGGAGAGACCCAGGAG	TTTCTGTAGACATCATCCTGGCTGG
**Cysltr1**	TCTTAAATTCACCATCTTCCTGCT	TCAGTTCCATTCATGTTCTCCA
**H2-Aa**	AAGACGACATTGAGGCCGA	AGTCCACCTTGGGGGTCAAA
**Hprt**	AGGGATTTGAATCACGTTTG	TTTACTGGCAACATCAACAG
**Lamp1**	ATTGCAGTTTGGGATGAATG	TTGCACTTGTATGAGTTTCC
**Lamp2**	GATCACGATGTGCCTCTCTC	GCAAGTACCCTTTGAATCTGTC
**Map1lc3b**	GCTCATCAAGATAATCAGACG	GCATAAACCATGTACAGGAAG
**Nup62**	GAATATCCCAGTGTCAAACC	AAACATTTTGATCAGGGACC
**Prkcd**	AAGCCCAAAGTGAAATCCCC	TCACAAAGGAGAAGCCATGGAA
**Rpl27**	GGACGCTACTCCGGACGCAAA	CCAAGGGGATATCCACAGAGTACC
**Sdha**	CTGTTATTGCTACTGGGGGCT	TACCTGTGGGGTGGAACTGA
**Sgk1**	TGCCAGCAACACCTATGC	GGACCCAGGTTGATTTGTTGA
**Sirt1**	TCCTTCAGTGTCATGGTTCCT	GGCTTCATGATGGCAAGTGG
**Sqstm1**	AAGAATGTGGGGGAGAGTGTG	GGAACTTTCTGGGGTAGTGGG
**Trem2**	GACCTCTCCACCAGTTTCTC	GCTTCAAGGCGTCATAAGTACA
**Tspo**	ACTTTGTACGTGGCGAGGG	ACTATGTAGGAGCCATACCCCA

### Immunofluorescence analysis

Fixed mouse retinas were dissected and quartered. Retinal whole mounts were washed with 50 mM Tris-buffered saline (TBS) and incubated in binding buffer (50 mM TBS + 0.5% Triton X 100 + 1% bovine serum albumin (BSA)) + 5% donkey serum overnight at RT to block nonspecific binding sites. Afterward, the tissues were washed 3 times with TBS for 20 minutes and incubated in binding buffer containing specific antibodies against GFAP (1:500, GP52, Progen, Germany), Iba1 (1:300, 019-19741, FUJIFILM Wako Pure Chemical Corporation, Japan), NG2 (1:300, 481 005, Synaptic Systems, Germany), ColIV (1:300, AB769, Merck Millipore, MA, USA), Brn3a (1:200, sc-31984, Santa Cruz Biotechnology, TX, USA), Sqstm1 (1:100, #5114, Cell Signaling Technology, UK), cathepsin D (1:100, ab75852, Abcam, UK), Lamp1 (1:50, 18992, Santa Cruz), Lamp2a (1:200, ab18528, Abcam) and γH2AX (1:200, 9718, Cell Signaling) for 3 days at 4°C. Whole mounts were washed 3 times with TBS for 60 minutes and further incubated in binding buffer containing Alexa Fluor 488-, Alexa Fluor 555- or Alexa Fluor 647-tagged donkey sera (1:1000, Thermo Fisher, MA, USA) to visualize the primary antibodies and 40,6-diamidino-2-phenylindole (DAPI, 1:4000) to visualize the cell nuclei overnight at RT. The retinas were subsequently washed 3 times with TBS for 20 minutes and embedded in TBS-glycerol (1:1). Secondary antibody-only controls were incubated in the absence of primary antibodies.

### Documentation and analysis

Fluorescence was documented by a confocal laser-scanning unit (AxioObserver Z1 attached to an LSM710, Zeiss, Germany; 20× dry or 63× oil immersion objective lenses, numerical aperture 1.30, Zeiss). Single optical section mode was used for image acquisition with appropriate filter settings for DAPI (345 nm excitation), Alexa Fluor 488 (495 nm excitation), Alexa Fluor 555 (509 nm excitation), and Alexa Fluor 647 (577 nm excitation). To determine the GFAP^+^ area, Iba1^+^ cell count, NG2^+^ cell count per capillary mm, ColIV^+^ structures and Brn3a^+^ cell count in specific retinal areas (superficial-deep or central-medial-peripheral), three images were captured per area to generate a mean value. For Sqstm1, cathepsin D, Lamp1, Lamp2a and γH2AX analysis, at least four images were randomly captured within the medial retinal region, focusing on the GCL and INL. For GCL images, the confocal laser-scanning-microscope (LSM) was focused on RGC nuclei and for INL images, the LSM was focused on the center of the INL along the Z axis of the tissue. Images of the GCL with a cell density of 80–120 cells/0.019 mm^2^ were used for statistical analysis. Further analysis was performed using ImageJ. To count Brn3a^+^ cells, images were smoothed and analyzed using a nucleus counter. Iba1^+^ cells were manually counted. Iba1^+^ and Brn3a^+^ cells were represented as counts/0.18 mm^2^. The GFAP^+^ area was determined by the default thresholding method and represented as the percentage of positive cells in the captured image (0.18 mm^2^). The diameter of the capillaries (<10 µm) was measured every ~20–30 µm. To analyze retinal vascularity, ColIV^+^ structures were smoothed, segmented by the default thresholding method, skeletonized and analyzed for total length, branch length and count, and vascular junctions. To determine retinal capillaries in the deep layer, ColIV^+^ structures were manually recolored to increase the signal-to-noise ratio. NG2^+^ cells were manually counted (image area: 0.18 mm^2^) and are represented as pericytes/capillary mm. The Sqstm1, cathepsin D, Lamp1, Lamp2a and γH2AX areas in the GCL and INL were determined by the default (Sqstm1) and triangle (cathepsin D, Lamp1, Lamp2a and γH2AX) thresholding methods.

### Proteasome activity

Snap-frozen quartered retinas were transferred to 100 µl of lysis buffer (50 mM Tris, 5 mM EDTA, 150 mM NaCl and 1% Triton X-100; pH 7.5) and vortexed. The tissues were incubated on ice for 30 minutes and vortexed every 10 minutes. Afterward, the lysates were centrifuged (12000 × g) for 15 minutes at 4°C. The supernatants were transferred to new tubes, and the protein concentrations were measured using a nanophotometer (Implen, Germany). Protein lysates (8 µg) were diluted in lysis buffer to a total volume of 100 µl containing 100 µM ATP and 600 µM substrate (Z-Gly-Gyl-Leu-AMC, Thermo Fisher). The mixtures were transferred to a black 96-well plate, and the fluorescence of the liberated AMC (Ex: 345/Em: 445) was detected using a DTX880 Multimode Detector (Beckman Coulter, CA, USA) [55]. The proteasome inhibitor MG-132 (100 µM, Thermo Fisher) was used as an assay control. The fluorescence intensity of free AMC was measured every 15 minutes between 0 and 120 minutes at RT. The blank value and basal substrate fluorescence (sample without protein) were subtracted from all values. The values of each sample were baseline corrected by subtracting the lowest value within the time response. The mean of two independent experiments was used for statistical calculations.

### Statistical analysis

GraphPad Prism 10.1.2 (GraphPad Software, Inc., CA, USA) was used to perform the statistical analysis. The applied statistical tests are specified in each figure legend. A *p*-value < 0.05 was considered to indicate statistical significance.

### Availability of data and materials

The datasets used and/or analyzed during the current study are available from the corresponding author upon reasonable request.
